# A small RNA decreases the sensitivity of *Shigella sonnei* to norfloxacin

**DOI:** 10.1186/s13104-019-4124-4

**Published:** 2019-02-21

**Authors:** I-Ning Gan, Hock Siew Tan

**Affiliations:** 1 School of Science, Monash University Malaysia, Jalan Lagoon Selatan, 47500 Subang Jaya, Selangor Malaysia; 2 Tropical Medicine & Biology Multidisciplinary Platform, Monash University Malaysia, Jalan Lagoon Selatan, 47500 Subang Jaya, Selangor Malaysia

**Keywords:** Small RNA, SdsR, Antibiotics resistance, *Shigella sonnei*, Norfloxacin, Fluoroquinolone

## Abstract

**Objectives:**

*Shigella* is a human pathogen that causes shigellosis, an acute invasive intestinal infection. Recent studies in the model bacterium *Escherichia coli* (*E. coli*) provided evidence that small regulatory RNAs (sRNAs) can contribute to antimicrobial resistance or susceptibility. One of the sRNAs is SdsR, which increases sensitivity of *E. coli* against fluoroquinolone by repressing the drug efflux pump, TolC. However, no reports exist about the effect of SdsR on fluoroquinolone resistance in *Shigella sonnei* (*S. sonnei*). In this study, we established the effect of SdsR on the sensitivity of *S. sonnei* to norfloxacin.

**Data description:**

We tested the effects of SdsR and SdsRv2 on fluoroquinolone resistance in *S. sonnei* in vivo. SdsRv2 is a synthetic version which promotes higher binding stability to *tolC* mRNA. Overexpression of either SdsR or SdsRv2 lowers the expression of *tolC* mRNA. Interestingly, SdsR and SdsRv2 promote the growth of *S.* *sonnei* in the presence of a sub-inhibitory concentration of norfloxacin. Mutant carrying SdsRv2 showed the highest growth advantage. This phenotype is opposite to the effect of SdsR reported in *E.* *coli*. This study is an example that demonstrates the difference in the phenotypic effect of a highly conserved sRNA in two closely related bacteria.

## Objective

The predominant *Shigella* species worldwide is *S. sonnei*, a less virulent but widely distributed across developed countries [1]. In the two last decades, *Shigella* have acquired resistance to many antibiotics, prompting the World Health Organization to list *Shigella* as a pathogen which urgently needs new antibiotics. One of the mechanism is through active efflux of fluoroquinolones [2]. These efflux pumps export antibiotics that accumulate in the cell, which enables the bacteria to survive antibiotic treatment. Bacteria often employ sRNAs as a post-transcriptional regulator of gene expression in response to various environmental challenges such as pH, temperature and antibiotics [3]. A sRNA known as SdsR regulates the expression of TolC, an efflux pump that promotes resistance to fluoroquinolone, a commonly prescribed antibiotic used to treat bacterial infections [4]. In *E. coli*, overexpression of SdsR decreases mRNA and protein levels of TolC [4] leading to an increase in sensitivity to fluoroquinolones [5].

Although *S. sonnei* is a close phylogenetic relative of *E. coli* [6], it is not clear whether SdsR plays a similar role in *S. sonnei*. Given the high conservation of SdsR and its target *tolC* in both *E. coli* and *S.* *sonnei*, we postulated that SdsR might perform a similar function in *S. sonnei.* We further hypothesized that increasing the stability of the RNA–RNA complex between SdsR and *tolC* mRNA may lead to an increase in the susceptibility of *S. sonnei* to norfloxacin due to downregulation of *tolC* mRNA. This study aims to determine the efficacy of SdsR and SdsRv2 in reducing the antibiotics resistance in *Shigella sonnei*.

## Data description

### Results

To increase the stability of RNA-RNA complex between SdsR and *tolC*, we incorporated four point mutations at the binding site of *tolC* in the design of SdsRv2 (Table 1, Data file 1). These mutations occurred in the predicted single-stranded loop region of SdsR. The native SdsR and artificially-designed SdsRv2 were overexpressed using the arabinose-inducible promoter system (Table 1, Data file 2). Semi-quantitative real-time PCR confirmed overexpression of both SdsR and SdsRv2 relative to the control strain (Table 1, Data file 3 and Table 1, Data file 4). The expression of *tolC* decreased in SdsR and SdsRv2 mutants respectively. The minimum inhibitory concentration (MIC) of norfloxacin in wild-type, SdsR and SdsRv2 mutants were determined to be at 0.06 μg/ml, 0.06 μg/ml and 0.09 μg/ml respectively. Since MIC only provides the endpoint measure but not information on growth kinetics, we monitored the growth curve of these mutants under two sub-inhibitory concentrations (0.02 μg/ml and 0.04 μg/ml) of norfloxacin. The SdsR and SdsRv2 mutants showed improved growth compared to the wild-type in the presence of 0.04 μg/ml norfloxacin (Table 1, Data file 5 and Table 1, Data file 6). The SdsRv2 mutant which have higher predicted binding stability to *tolC* mRNA showed the highest growth rate compared to other strains. To our knowledge, this is the first report to show that although *tolC* mRNA was down-regulated by SdsR and SdsRv2, the sensitivity against norfloxacin decreased in *S. sonnei*.

**Table 1:** Overview of data files

Label	Name of data file/data set	File types	Data repository and identifier
Data file 1 [7]	Design of synthetic RNA, SdsRv2	PDF	*Figshare* (10.6084/m9.figshare.7429040)
Data file 2 [8]	Materials and method details	DOCX	*Figshare* (10.6084/m9.figshare.7429043)
Data file 3 [9]	Expression levels of SdsR and *tolC*	PDF	*Figshare* (10.6084/m9.figshare.7429034)
Data file 4 [10]	Raw data used to generate graph in data file 3	XLSX	*Figshare* (10.6084/m9.figshare.7483538)
Data file 5 [11]	Growth curve of different mutants	PDF	*Figshare* (10.6084/m9.figshare.7429037)
Data file 6 [12]	Raw data used to generate graphs in data file 5	XLSX	*Figshare* (10.6084/m9.figshare.7483562)

## Limitations

The shortcomings of this paper that prevented the data to be published in a regular paper are:

- SdsRv2 was tested in an *S. sonnei* strain that still maintains the wild-type copy of the SdsR. Although SdsRv2 should be able to compete with native SdsR for binding to its targets, the sole effect of SdsRv2 cannot be clearly defined when both species of RNA are present in a single cell.
- The effect of SdsR on antibiotics resistance in *S. sonnei* contradicts the phenotype observed in *E.* *coli*. Elucidation of the mechanism behind this phenotype requires further study, which is beyond the scope of this project. For example, a translational fusion of the *tolC* UTR (untranslated region) to a reporter gene can be used to establish SdsR regulation. Nevertheless, this study presented an interesting contradictory result. The results from this project are being considered by the authors for future research to elucidate the reason for such discrepancy.

## Abbreviations

sRNA: small RNA
MIC: minimum inhibitory concentration
